# Combined strategies for improving expression of *Citrobacter amalonaticus* phytase in *Pichia pastoris*

**DOI:** 10.1186/s12896-015-0204-2

**Published:** 2015-09-26

**Authors:** Cheng Li, Ying Lin, Xueyun Zheng, Nuo Pang, Xihao Liao, Xiaoxiao Liu, Yuanyuan Huang, Shuli Liang

**Affiliations:** Guangdong Key Laboratory of Fermentation and Enzyme Engineering, School of Bioscience and Bioengineering, South China University of Technology, Guangzhou, 510006 P. R. China; Guangdong Research Center of Industrial Enzyme and Green Manufacturing Technology, School of Bioscience and Bioengineering, South China University of Technology, Guangzhou, 510006 P. R. China

## Abstract

**Background:**

Phytase is used as an animal feed additive that degrades phytic acid and reduces feeding costs and pollution caused by fecal excretion of phosphorus. Some phytases have been expressed in *Pichia pastoris*, among which the phytase from *Citrobacter amalonaticus* CGMCC 1696 had high specific activity (3548 U/mg). Improvement of the phytase expression level will contribute to facilitate its industrial applications.

**Methods:**

To improve the phytase expression, we use modification of P_*AOX1*_ and the α-factor signal peptide, increasing the gene copy number, and overexpressing *HAC1*^*i*^ to enhance folding and secretion of the protein in the endoplasmic reticulum. The genetic stability and fermentation in 10-L scaled-up fed-batch fermenter was performed to prepare for the industrial production.

**Results:**

The phytase gene from *C. amalonaticus* CGMCC 1696 was cloned under the control of the *AOX1* promoter (P_*AOX1*_) and expressed in *P. pastoris*. The phytase activity achieved was 414 U/mL. Modifications of P_*AOX1*_ and the *α*-factor signal peptide increased the phytase yield by 35 and 12 %, respectively. Next, on increasing the copy number of the Phy gene to six, the phytase yield was 141 % higher than in the strain containing only a single gene copy. Furthermore, on overexpression of *HAC1*^*i*^ (*i* indicating induced), a gene encoding Hac1p that regulates the unfolded protein response, the phytase yield achieved was 0.75 g/L with an activity of 2119 U/mL, 412 % higher than for the original strain. The plasmids in this high-phytase expression strain were stable during incubation at 30 °C in Yeast Extract Peptone Dextrose (YPD) Medium. In a 10-L scaled-up fed-batch fermenter, the phytase yield achieved was 9.58 g/L with an activity of 35,032 U/mL.

**Discussion:**

The production of a secreted protein will reach its limit at a specific gene copy number where further increases in transcription and translation due to the higher abundance of gene copies will not enhance the secretion process any further. Enhancement of protein folding in the ER can alleviate bottlenecks in the folding and secretion pathways during the overexpression of heterologous proteins in *P. pastoris*.

**Conclusions:**

Using modification of P_*AOX1*_ and the *α*-factor signal peptide, increasing the gene copy number, and overexpressing *HAC1*^*i*^ to enhance folding and secretion of the protein in the endoplasmic reticulum, we have successfully increased the phytase yield 412 % relative to the original strain. In a 10-L fed-batch fermenter, the phytase yield achieved was 9.58 g/L with an activity of 35,032 U/mL. Large-scale production of phytase can be applied towards different biocatalytic and feed additive applications.

**Electronic supplementary material:**

The online version of this article (doi:10.1186/s12896-015-0204-2) contains supplementary material, which is available to authorized users.

## Background

Phytases (*myo*-inositol hexakisphosphate 3- or 6-phosphohydrolases; EC 3.1.3.8 or EC 3.1.3.26) are phosphatases that catalyze the stepwise removal of phosphate from phytate. The addition of phytase to feed for monogastric animals increases phosphorus availability [1], reducing feeding costs by reducing the requirement for supplementation with inorganic phosphorus [2] and the pollution caused by fecal excretion of phosphorus [3]. Most phytases are produced in fungi and bacteria, but only some of them can be expressed at a high level, such as with the *Escherichia coli* phytase gene *appA* expressed in *Pichia pastoris* (118 to 204 U/ml at the flask scale) [4], *Aspergillus niger* phytase in *A. niger* (1008 U/g in flasks) [5], and *Citrobacter amalonaticus* CGMCC 1696 phytase (420 U/mL in flasks) expressed in *P. pastoris* [6]. Among these, the phytase from *C. amalonaticus* CGMCC 1696 has the highest specific activity, 3548 U/mg [6]. The *C. amalonaticus* phytase expressed by *P. pastoris* also has an optimal temperature of 60 °C and pH of 3.0 [7], which meets the requirements of industrial applications. However, the high cost of phytase remains a barrier to its use in industrial applications. To facilitate the use of *C. amalonaticus* phytase in industry, high-level expression in an industrial-grade host is necessary.

*P. pastoris* has become an important tool, especially for heterologous protein production [8]. This methylotrophic yeast is a valuable production system because of its ability to grow to very high cell densities, its ability to produce gram amounts of recombinant proteins per liter of culture both intracellularly and in secretory fashion, and because of the availability of the strong and tightly regulated promoter *AOX1* (P_*AOX1*_) [9]. The promoter type affects transcription efficiency. Moreover, novel artificial promoters can be assembled by combining cis-acting elements with P_*AOX1*_ to improve the expression of heterologous protein [10]. The *Saccharomyces cerevisiae α*-factor prepro-signal, consisting of a 19-amino acid pre-region signal peptide and a pro-region of 60 hydrophilic amino acids [11], is the most widely and successfully used secretion signal peptide in *P. pastoris*. Optimizing the structure of the *α*-factor [12, 13] and choosing endogenous signal peptides, such as the signal peptides of Dse4p [14], may enhance the export of heterologous protein expressed in *P. pastoris*. Moreover, gene copy number also influences the expression level efficiency [15].

In the natural state, if the expression vector transformed into *P. pastoris* is a single copy, the probability of the emergence of multi-copy integration is about 10 % and the copy number is not controllable [16]. Therefore, several high-throughput methods have been established to screen a large number of clones for multi-copy integration based on small-scale cultivation [17–19]. Sometimes, high copy number can even be detrimental for recombinant protein productivity [20–22]. Furthermore, high expression of heterologous proteins may cause increasing accumulation of misfolded protein, which then causes endoplasmic reticulum (ER) stress and activates the unfolded protein response (UPR) [23]. Folding and secretion of protein in the ER may be the rate-limiting step in the secretion of a heterologous protein [24, 25]. To overcome these problems, strategies to improve the folding and secretion of proteins have been investigated. Such approaches involved overexpressing the active transcription factor of UPR target gene *HAC1*^*i*^ (*i* indicating induced) [26–28], enhancing the expression of the ER chaperone BiP (Kar2p) [29], and increasing expression of disulfide isomerase (Pdi1p) [30] and endoplasmic reticulum oxidoreductin 1 (Ero1p) [31].

In this study, we used a series of combined genetic modification strategies to enhance production of phytase from *C. amalonaticus* CGMCC 1696 (Phy) in *P. pastoris*. These strategies involved modification of P_*AOX1*_, choosing an appropriate signal peptide, and augmentation of the gene dose of AOX1-driven Phy by head-to-tail cassette multimerization. ER stress limits the production of heterologous proteins [32, 33], thus we overexpressed the transcription factor Hac1p and the chaperones Pdi1p, Kar2p and Ero1p.

## Methods

### Strains and growth conditions

*Escherichia coli* TOP10F’ (Invitrogen, Carlsbad, CA, USA) cells were used for DNA manipulations; these cells were cultivated in LB or low-salt LB medium. Bacterial plasmid selection and maintenance was performed using 100 mg/L kanamycin or 25 mg/L zeocin (Invitrogen). The *P. pastoris* strain GS115 (Invitrogen) was used as the host cell, and this strain was cultivated in YPD medium (1 % yeast extract, 2 % peptone, and 2 % glucose). Transformants of *P. pastoris* were selected on MD (1.34 % YNB, 4 × 10^−5^% biotin, 2 % dextrose, and 2 % agar) or YPDSZ plates (1 % yeast extract, 2 % peptone, 2 % glucose, 18.2 % sorbitol, 2 % agar, and 100 mg/L zeocin). The trace metal solution PTM1 (CuSO_4_ · 5H_2_O 0.6 %, CoCl_2_ 0.05 %, FeSO_4_ · H_2_O 6.5 %, KI 0.009 %, H_3_BO_3_ 0.002 %, H_2_SO_4_ 0.5 %, MnSO_4_ · H_2_O 0.3 %, MoNa_2_O_4_ · 2H_2_O 0.024 %, ZnCl_2_ 2 %, and biotin 0.02 %) and basal salt medium (BSM) (glycerol 4 %, CaSO_4_ 0.093 %, K_2_SO_4_ 1.82 %, KOH 0.413 %, MgSO_4_ · 7H_2_O 1.49 %, H_3_PO_4_ 2.67 %, and PTM1 0.435 %) were used in fed-batch cultivation. A 50 % (*w/v*) glycerol solution containing 1.2 % (*v/v*) PTM1 and methanol containing 1.2 % (*v/v*) PTM1 were used as feed solutions according to the protocol of the *“Pichia Fermentation Process Guidelines”* (Invitrogen).

The phytase gene of *C. amalonaticus* CGMCC 1696, *PHY* [GenBank: ABI98040.1], was from the vector pPICZαA-phy [7]. The vectors pPICHKA and pPICZA-HAC1 were gifts from Dr. Han (South China University of Technology) [22]. The plasmid pTEFZA-EGFP-HIS-G, which contained the *GAPDH* gene fragment, was from a previous study [14]. Strains, vectors and primers used in the present study are summarized in Additional file 2.

### Construction of vectors

Plasmid construction is illustrated in Additional file 1. The combining of cis-acting elements with P_*AOX1*_ used the same methods as described by Hartner et al. [10]: 5′ arms of P_*AOX1*_ were amplified by PCR using primer pairs AOX F and D1 R or AOX F and 201-2xrv, and the 3′ arms using primer pairs AOX R and D1 F or AOX R and 201-2xfw. The P_*AOX1*_ variants were generated using primer pairs AOX F and AOX R (Additional file 2, the *Bgl*II and *Bst*BI sites are underlined), resulting in the vector pAOX1_d1+201_-α-phy. To create the plasmids pPICHKA-phy (Phy) and pAOX1_d1+201_-α-phy-HKA (AOXm), the HIS4 and kanamycin resistance genes from plasmid pPICHKA (HKA) were used to replace the zeocin resistance gene in the plasmids pPICZαA-phy and pAOX1_d1+201_-α-phy using *Bam*HI and *Mlu*I sites.

The signal peptide of Dse4p was obtained from *P. pastoris* strain GS115 genomic DNA using the primer pair SP-D1 and SP-D2 (Additional file 2, the *Bst*BI and *Eco*RI sites are underlined). The modified *α*-factor with a 10-residue spacer peptide [12] was amplified by PCR using the primer pair SP-M1 and SP-M2 (Additional file 2, the *Bst*BI and *Eco*RI sites are underlined). The modified α-factor with a deletion of the predicted third alpha helix was amplified by overlapping PCR: the primer pair SP-M1 and SP-∆57-70-2 were used to produce fragment ∆57-70-SP1, and the primer pair SP-∆57-70-1 and SP-∆2 were used to generate fragment ∆57-70-SP2. Then, the fragment ∆57-70-SP was amplified by PCR using the fragments ∆57-70-SP1 and ∆57-70-SP2 as template with the primer pair SP-M1 and SP-∆2 (Additional file 2, the *Bst*BI and *Eco*RI sites are underlined). All the PCR products were used to replace the *α*-factor gene in the plasmid pAOX1_d1+201_-α-phy, resulting in the vectors pAOX1_d1+201_-D-phy, pAOX1_d1+201_-αE10-phy, pAOX1_d1+201_-α∆57-70-phy. Then the zeocin resistance gene in these plasmids was replaced by the HIS4 and kanamycin resistance genes from plasmid pPICHKA using BamHI and MluI sites to create vectors pAOX1_d1+201_-D-phy-HKA (SP-D), pAOX1_d1+201_-αE10-phy-HKA (αE10), and pAOX1_d1+201_-α∆57-70-phy-HKA (α∆57-70).

Digestion of pPICZA-αE10-phy-HKA with *Bgl*II (Thermo Scientific, Waltham, MA, USA) and *Bam*HI (Thermo Scientific) releases the expression cassette (PAOX1_d1+201_ plus Phy gene, approximately 2700 bp, Additional file 3). After production of the *Bgl*II-*Bam*HI expression cassette fragment and linearization of pPICZA-αE10-phy-HKA using *Bam*HI, the insert and the plasmid were ligated to obtain the two-copy plasmid pPICZA-αE10-HKA/(Phy)_2_ (2c). A similar method was used to obtain the four-copy and six-copy plasmids, pPICZA-αE10-HKA/(Phy)_4_ (4c) and pPICZA-αE10-HKA/(Phy)_6_ (6c), respectively. The *GAPDH* gene fragment from the plasmid pTEFZA-EGFP-HIS-G was ligated into the plasmid pPICZαA-phy using the *Apa*I-*Not*I sites to create the vector pPICZαA-phy-G. The genes *PDI* [GenBank: ACF17572.1], *KAR2* [GenBank: AAX77226.1] and *ERO1* [GenBank: CAY67364.1] were obtained from *P. pastoris* strain GS115 genomic DNA using appropriate primer pairs (Additional file 2, the *Pml*I and *Sac*II sites are underlined). All the PCR products were ligated into the plasmid pPICZA (Invitrogen) using the *Pml*I-*Sac*II sites to create vectors pPICZA-PDI, pPICZA-KAR2, and pPICZA-ERO1. Restriction enzyme digestion and DNA sequencing assured that all plasmids matched their designs (data not shown).

### Yeast transformation

Plasmids HKA, Phy, AOXm, SP-D, αE10, α∆57-70, 2c, 4c, and 6c were linearized with *Kpn*2I (Thermo Scientific), whose cleavage site was located in the *his4* sequence, and transformed into *P. pastoris* GS115 competent cells using the standard lithium chloride transformation method according to the manufacturer’s protocol (Invitrogen). The transformed cells were selected on MD plates and incubated at 30 °C for 3 days. Recombinants of GS115/αE10 were selected on plates with MD medium containing G418 at 1, 2, 5, 7, and 9 mg/mL. The integration of the recombinant plasmid, αE10, into the GS115 genome was verified by colony PCR using 5′AOX1 and 3′AOX1 as the sequencing primers (data not shown).

The plasmids pPICZA-HAC1, pPICZA-PDI, pPICZA-KAR2, and pPICZA-ERO1 were linearized with *Mss*I (Thermo Scientific), whose cleavage site was located in the *AOX1* promoter sequence, and transformed into GS115/6c competent cells. The transformed cells were selected on YPDSZ plates and incubated at 30 °C for 3–4 days. The integration of the recombinant plasmids into the GS115/6c genome was verified by colony PCR using 5′AOX1 and HAC-A, PDI-2, KAR2-2, or ERO-2 as the sequencing primers (data not shown).

### Determination of the *phy* copy number by quantitative PCR

The quantitative PCR (qPCR) assay protocol was derived from the Pfaffl method [34]. The plasmid pPICZαA-phy-G, consisting of a portion of the *GAPDH* gene and the following genomic sequence, was used as the reference sequence because there is only a single copy in the *P. pastoris* genome [35]. Genomic DNA was extracted from *P. pastoris* using the Yeast DNAiso-Kit (Takara, Shiga, Japan) according to the manufacturer’s manual. RT-Phy1/RT-Phy2 and RT-G1/RT-G2 were used as primers at concentrations of 400 nM with genomic DNA as the template. The yeast recombinant DNA and the standard plasmid were analyzed simultaneously using SYBR®Premix Ex *Taq*™ II (Tli RNaseH Plus) (Takara) in an Applied Biosystems®7500 fast real-time PCR instrument (Applied Biosystems Inc., Foster City, CA, USA). The qPCR assay was repeated three times for one sample. The copy number of *phy* in each transformant was calculated using the Ct value of the *P. pastoris* genomic DNA and a standard curve. The Phy gene copy number in the *P. pastoris* genome was determined with the following equation:

$$ phy\ \mathrm{gene}\ \mathrm{copy}\ \mathrm{number}=\frac{phy\ \mathrm{gene}\ \mathrm{copy}\ \mathrm{number}\ \mathrm{calculated}\ \mathrm{b}\mathrm{y}\ \mathrm{standard}\ \mathrm{curve}}{\mathrm{GPADH}\ \mathrm{fragment}\ \mathrm{copy}\ \mathrm{number}\ \mathrm{calculated}\ \mathrm{b}\mathrm{y}\ \mathrm{standard}\ \mathrm{curve}} $$

### Cultivation of *P. pastoris* and expression of phytase

*P. pastoris* transformants were inoculated into 5 mL of BMGY medium (1 % yeast extract, 2 % peptone, 1.34 % YNB, 0.00004 % biotin, 100 mM potassium phosphate (pH 6.0), and 1 % glycerol) in a 50-mL Erlenmeyer flask. The cells were precultivated overnight at 30 °C and 250 rpm. Next, the main cultures were inoculated from precultures to obtain an initial optical density of 0.5. The cells were grown in 20 mL of BMMY medium (1 % yeast extract, 2 % peptone, 1.34 % YNB, 0.00004 % biotin, 100 mM potassium phosphate (pH 6.0), and 1 % methanol) in a 250-mL Erlenmeyer flask in a shaking incubator at 30 °C and 250 rpm. Fresh methanol was added to obtain a final concentration of 1 % (*v/v*) every 24 h. OD_600_ and phytase activity were monitored throughout a 5-day incubation.

The fermentation of the recombinant *P. pastoris* was performed in a 10-L standard fermenter (FUS10-A, Shanghai Guoqiang Bioengineering Equipment Co., Ltd., Shanghai, China) containing 5 L BSM. The cultivation parameters of the fermenter were as follows: growth temperature 30 °C, growth pH5.5, air flow rate 10 L/min, and stirring speed 800 rpm. Typical recombinant *P. pastoris* fermentation comprised three phases. The entire cultivation started with a batch phase (phase I) in BSM for initial cell growth; this phase lasted for about 18–24 h at 30 °C and pH5.5. After the glycerol in the medium was exhausted, the fed-batch phase (phase II) was initiated by feeding limited glycerol to allow further cell growth. When OD_600_ reached approximately 230, glycerol feeding was ended and there was a carbon-source starvation period of 30 min to allow complete consumption of the glycerol and its mesostates. At the same time, the temperature was reduced to 25 °C and the pH of the broth was adjusted to 6.0 by adding ammonia solution (25 %, *v/v*). The induction phase (phase III) was started by the addition of 10–15 g/h mixtures of glycerol and methanol (100 % methanol: 50 % glycerol = 2:1, *v/v*) as carbon source. The mixture feed rate was then adjusted upwards every 1.5–2 h until reaching 40 ± 2 g/h, while dissolved oxygen (DO) was kept constant at about 20–30 %. OD_600_ and phytase activity were monitored throughout a 7-day induction.

### Phytase enzymatic activity

Phytase activity was analyzed according to the method described by Žyla [36], with modifications. Fifty microliters of culture were centrifuged for 1 min at 8000 rpm and room temperature. The supernatants were diluted with 100 mM sodium acetate buffer (pH5.5) to reach a volume of 1 mL, and the mixture was preheated at 37 °C for 5 min. Next, 2 mL of 5.0 mM sodium phytate (pH 5.5) was added and the mixture was incubated at 37 °C for 30 min, then 2 mL of coloration solution [24 % nitric acid, 100 g/L ammonium molybdate, and 2.35 g/L ammonium vanadate, 2:1:1 (v/v/v)] was added and incubated for 10 min. The absorbance of the mixture was measured at 415 nm. One unit of activity (U) was defined as the amount of enzyme that hydrolyzed 5.0 mM sodium phytate per min to generate 1 μmol of inorganic phosphorus at 37 °C. All values are averages obtained from three independent experiments and use GS115/HKA as background (control) samples.

### SDS-PAGE and protein content analysis

After induction, sodium dodecyl sulfate polyacrylamide gel electrophoresis (SDS-PAGE) was performed on culture supernatants using 12 % SDS-polyacrylamide gels. The supernatants of strain GS115/Phy were treated with PNGase F (NEB, Boston, MA, USA). Supernatant was heat-denatured by treating at 100 °C for 5 min in denaturing buffer containing 1 % SDS and 0.5 % 2-mercaptoethanol. The proteins were stained with Coomassie Brilliant Blue R-250 (Invitrogen).

The phytase concentration in the supernatants was estimated by SDS-PAGE with bovine serum albumin (BSA; Invitrogen) as the standard. The amount of Phy in supernatants expressed by *P. pastoris* was quantified with equal volumes of BSA of known concentrations as an external reference protein and the Phy band was analyzed using Quantity One software (Bio-Rad, CA, USA).

### Genetic stability of recombinant *P. pastoris*

To demonstrate the stable inheritance and stable expression levels of the phytase genes in progenies [37], clones of the strain 6c/HAC1 were grown on rich YPD selective medium for 10 cycles. After the first culture reached the stationary phase of growth on YPD medium, 1 % culture was inoculated into new 100-ml YPD medium for the next cycle of growth. This was repeated for ten cycles. Phytase activity and *PHY* copy number were determined in the strain after 10 sub-cultivations.

## Results

### Construction of phytase expression system

Plasmid construction in this work is illustrated in Additional file 1. The vector pPICZαA-phy [7], which carries the phytase gene of *C. amalonaticus* CGMCC 1696, had the zeocin resistance gene replaced by the HIS4 and kanamycin resistance genes from plasmid pPICHKA [22] to create plasmid pPICHKA-phy (Phy). The combination of cis-acting elements with P_*AOX1*_, a deletion of P_*AOX1*_ nucleotides −777 and −712 and the addition of −203 and −190 [10], was performed to create plasmid pAOX1_d1+201_-α-phy-HKA (AOXm). Based on this plasmid, a 10-residue spacer peptide (EEAEAEAEPK) between the *α*-factor prepro-signal [13] and the phytase gene was introduced to create plasmid pAOX1_d1+201_-αE10-phy-HKA (αE10); a deletion of the predicted third alpha helix of the *α*-factor [12] was performed to create plasmid pAOX1_d1+201_-α∆57-70-phy-HKA (α∆57-70); and replacement of the *α*-factor by the signal peptide of Dse4p [14] was undertaken to create plasmid pAOX1_d1+201_-D-phy-HKA (SP-D). Based on the plasmid αE10, plasmids pPICZA-αE10-HKA/(Phy)_2_ (2c), pPICZA-αE10-HKA/(Phy)_4_ (4c), and pPICZA-αE10-HKA/(Phy)_6_ (6c) were created, which contained two, four and six expression cassettes respectively (Additional files 1 and 3).

All plasmids were transformed into *P. pastoris* GS115 after linearization with *Kpn*2I; the resulting strains were designated GS115/HKA, GS115/Phy, GS115/AOXm, GS115/αE10, GS115/α∆57-70, GS115/SP-D, GS115/2c, GS115/4c and GS115/6c. The recombinant strains of GS115/αE10 were selected on MD plates supplemented with different concentrations of the aminoglycoside antibiotic G418 to obtain strains containing high copy numbers (GS115/nc). The plasmids pPICZA-HAC1, pPICZA-PDI, pPICZA-KAR2, and pPICZA-ERO1 were transformed into recombinant strain GS115/6c after linearization with *Mss*I to form strains 6c/HAC1, 6c/PDI, 6c/KAR2, and 6c/ERO1.

### Gene copy number determination by quantitative PCR

The copy number of target genes can significantly influence the production of recombinant protein, and neglecting gene copy number can easily lead to an incorrect interpretation of experimental results concerning codon optimization, promoter studies, co-expression of helper proteins, and signal peptide optimization [38]. Therefore, qPCR assays were performed to precisely determine the Phy gene copy number in the genomes of the integrants.

**Fig. 1:**
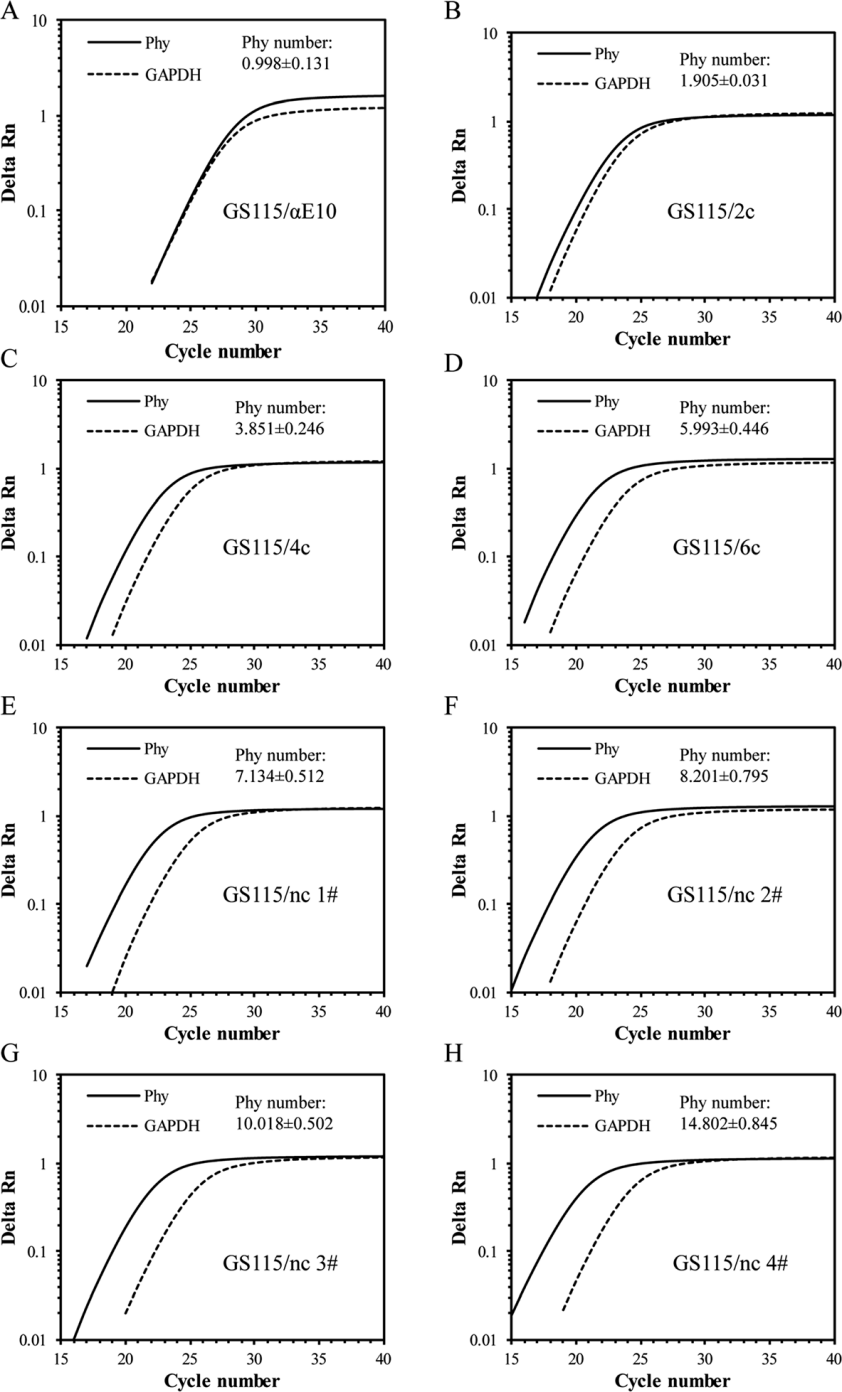
Quantitative PCR assay of the *PHY* copy number in recombinant yeast strain genomic DNA. The threshold value (*horizontal dashed line*) was set at 0.2. The values indicate the average ± standard deviations from three independent qPCR experiments

The Phy gene copy numbers of strains GS115/Phy, GS115/AOXm, GS115/αE10, GS115/α∆57-70, and GS115/SP-D, normalized to the reference *GAPDH* gene fragment [35], were 0.997, 0.989, 0.998, 0.982, and 0.984, respectively (Additional file 4). These results indicate that the strains above contained a single copy of the Phy gene, ruling out the influence of copy number on the secretion of Phy. The strains GS115/2c, GS115/4c, and GS115/6c were confirmed to contain two, four, and six copies of *PHY* respectively (Fig. 1). We also obtained some high copy number integrants under the pressure of G418, carrying seven, eight, ten, and 15 copies of the Phy gene (Fig. 1).

### Phy expression in *P. pastoris*

**Fig. 2:**
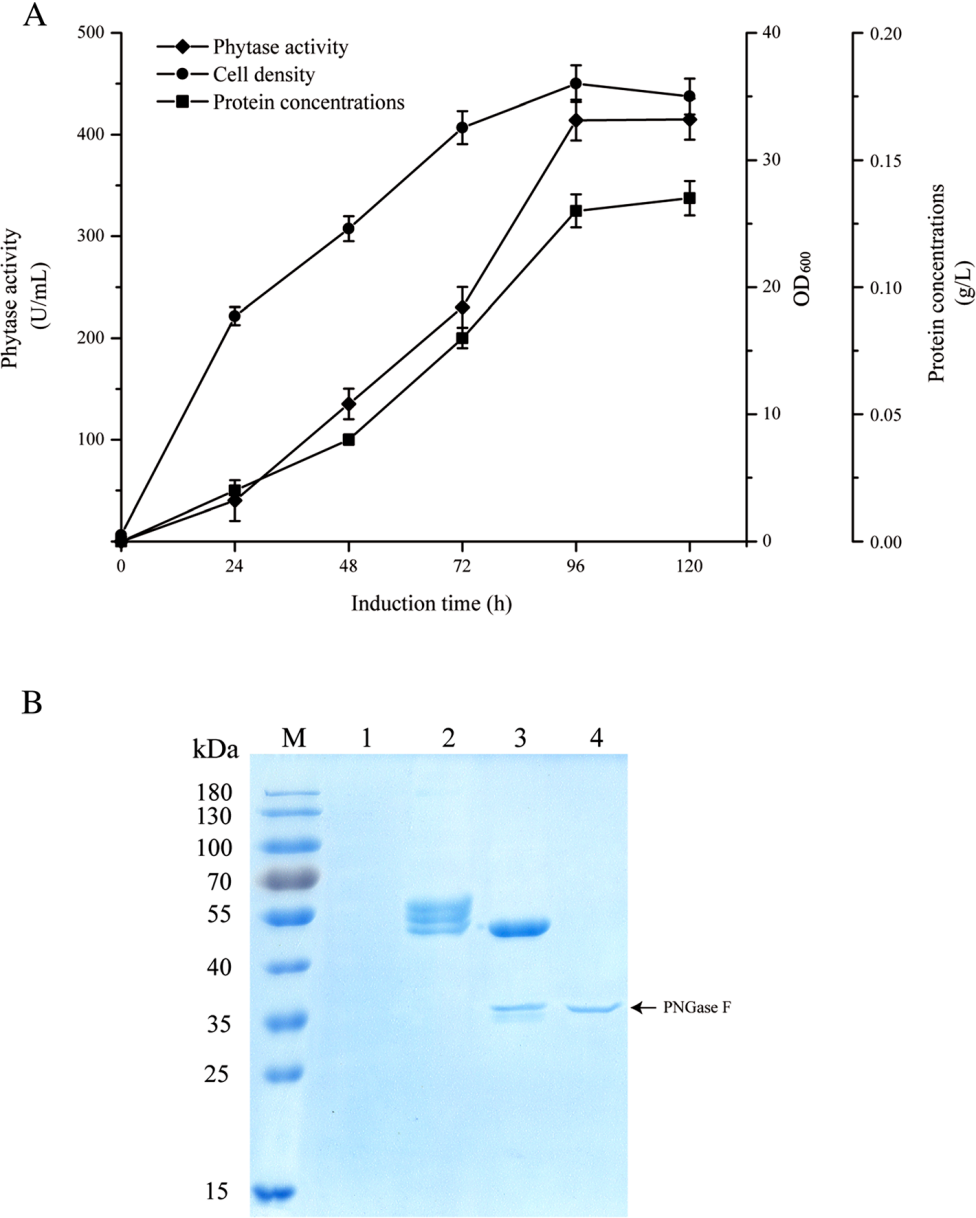
Expression of the phytase from *C. amalonaticus* CGMCC 1696 in *P. pastoris*. **a**: Time dependence of the activity of phytase, cell growth and phytase protein content of Gs115/Phy after induction with methanol, using GS115/HKA as the background (control) sample. **b**: SDS-PAGE assay of the culture supernatant containing *C. amalonaticus* CGMCC 1696 phytase (*stained with Coomassie Blue*) after methanol induction for 96 h. Lane 1: culture supernatants from recombinant *P. pastoris* GS115/HKA; lane 2: culture supernatants from recombinant *P. pastoris* Gs115/Phy; lane 3: culture supernatants from recombinant *P. pastoris* Gs115/Phy after PNGase F treatment; lane 4: culture supernatants from recombinant *P. pastoris* GS115/HKA after PNGase F treatment

After 96 h of induction with methanol, the phytase obtained reached the maximum observed level. The phytase activity and protein content of strain GS115/Phy were 414 U/mL and 0.13 g/L (Fig. 2a). Figure 2b shows that the molecular weight of recombinant phytase was approximately 50 kDa, assayed by SDS-PAGE. There were several bands visible on SDS-PAGE on separation of Gs115/Phy supernatants, but after PNGase F treatment there was only one band, of nearly 43 kDa, which was similar to that described by Luo et al. [6]. This suggested that the phytase protein was partially glycosylated in *P. pastoris* and the observed molecular weights were higher than the molecular weight predicted from the amino acid sequence alone (46.3 kDa).

### Combined strategies for enhancement of Phy expression in *P. pastoris*

**Fig. 3:**
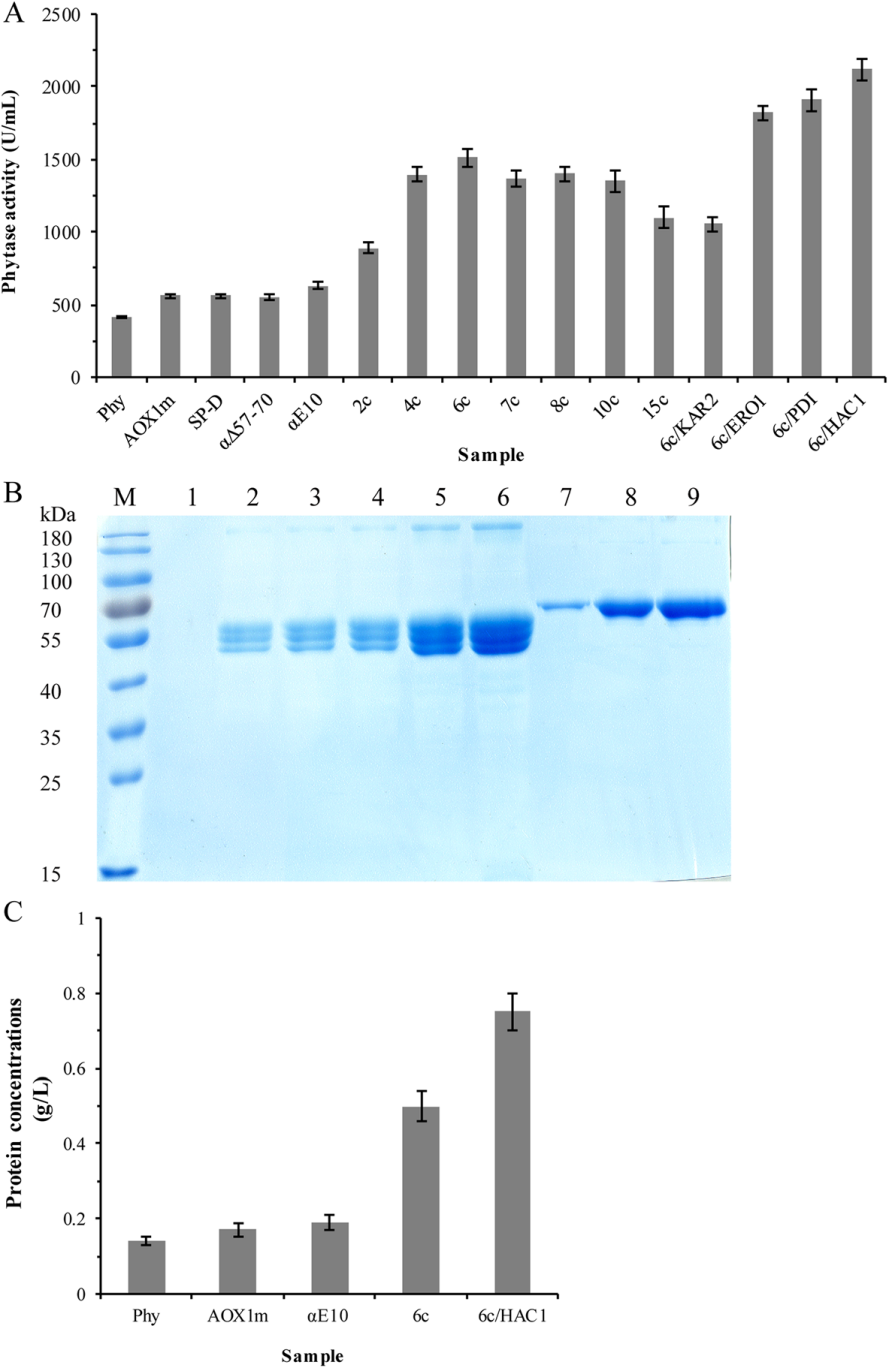
Different tactics for enhancing the expression of Phy in *P. pastoris*. **a**: Effect of modification of P_*AOX1*_ and the signal peptide, increasing gene copy number, and overexpression of Kar2p, Ero1p, Pdi1p, or Hac1p on phytase production in recombinant strains carrying six Phy gene copies after 96-h induction with methanol. All activities used GS115/HKA as the background measurement. **b**: SDS-PAGE assay of the collected culture supernatant from the most effective strains after 96-h induction with methanol. Lane M: protein marker; lane 1: GS115/HKA; lane 2: GS115/Phy; lane 3: GS115/AOXm; lane 4: GS115/αE10; lane 5: GS115/6c; lane 6: 6c/HAC1; lane 7: 0.05 mg/mL BSA; lane 8: 0.2 mg/mL BSA; lane 9: 0.3 mg/mL BSA. **c**: The phytase protein content of the most effective strains after 96-h induction with methanol

After 96 h of induction with methanol, the phytase obtained reached the maximum observed level for all recombinant strains (data not shown). Modification of the putative *cis*-acting region of P_*AOX1*_ nucleotides, i.e., deleting −777 and −712 and adding −230 and −190, resulted in an increase in phytase activity of about 35 %, reaching 560 U/mL (Fig. 3a), which was similar to the results of Hartner et al. [10].

Among all the modifications of the signal peptide, only the addition of a 10-residue spacer peptide (EEAEAEAEPK) between the *α*-factor prepro-signal and the phytase gene increased yield, by 12 % relative to that of the *α*-factor signal peptide alone, to 626 U/mL (Fig. 3a). 

In the high copy number integrants, Phy productivity increased with the gene copy number up to a maximum of six copies; the phytase activity of strain GS115/6c increased 141 % (reached 1511 U/mL, Fig. 3a) relative to the corresponding single-copy strains, but then there was a plateau effect when the copy number was increased further (Fig. 3a and Additional file 5).

On further modifying strain GS115/6c, overexpression of Pdi1p and Ero1p enhanced Phy secretion by 27 and 20.6 % (Fig. 3a), respectively. Overexpression of Hac1p improved phytase activity by 40 % relative to that for strain GS115/6c; it reached 2119 U/mL (Fig. 3a), which was similar to the effect observed when overexpression of Hac1p enhanced *Bacillus amyloliquefaciens* α-amylase secretion in *S. cerevisiae* [27] and secretion of xylanase A from *Bacillus halodurans* C-125 in *P. pastoris* [28].

On combination of all of the methods used above to improve Phy expression, the phytase activity and protein content of strain 6c/HAC1 finally reached 2119 U/mL (Fig. 3a) and 0.75 g/L (Fig. 3b and c) in 250-mL shaken flasks, an increase of 412 % relative to the original strain GS115/Phy.

### Phytase production by the recombinant strains in a bench-top fermenter

**Fig. 4:**
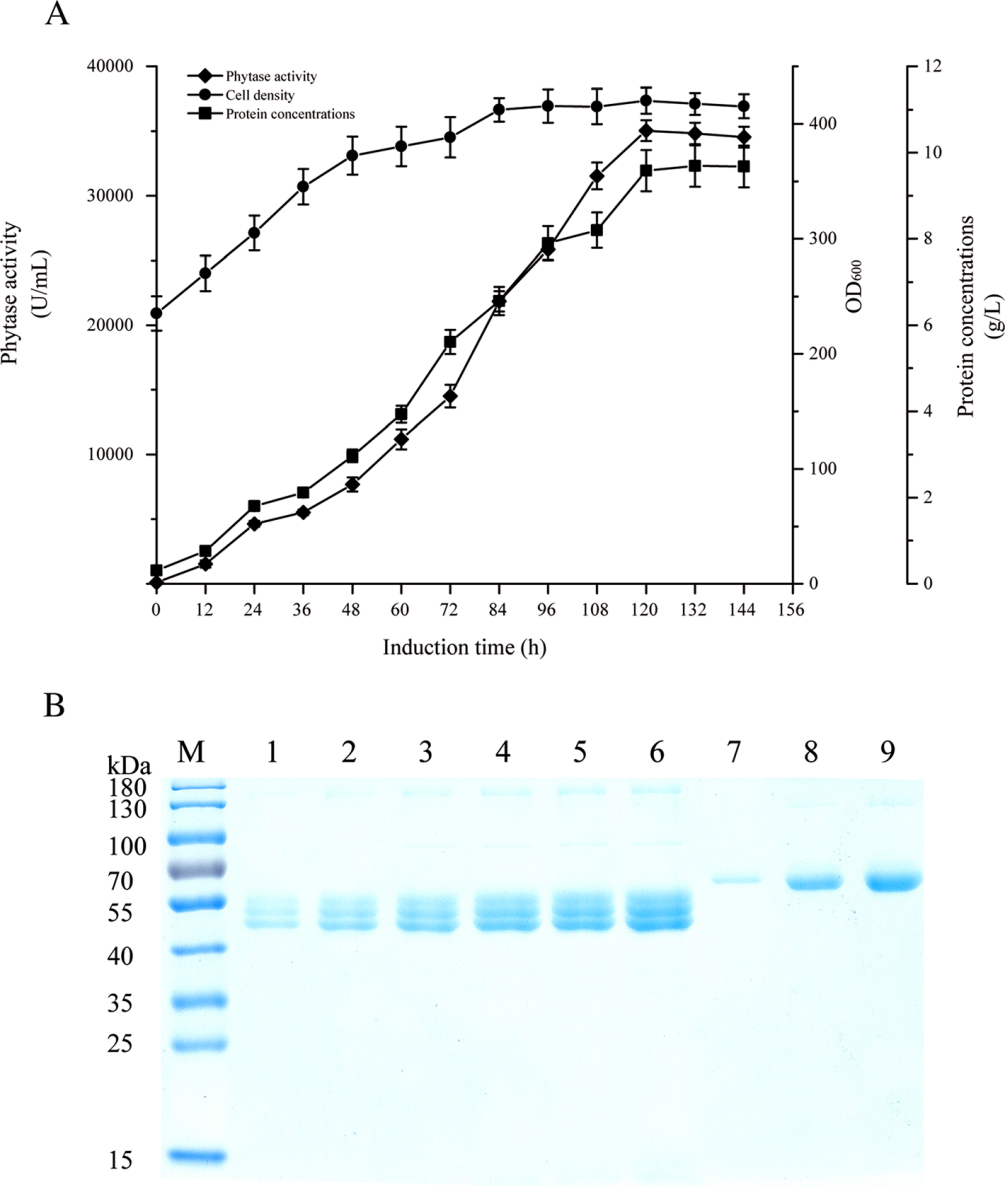
Growth and phytase production time course of *P. pastoris* 6c/HAC1 in a 10-L fermenter. **a**: Time dependence of phytase activity, cell density and phytase protein content after induction with methanol. The phytase activity was detected using collected culture supernatant. The sample was boiled for 10 min was used as the background sample. **b**: SDS-PAGE assay of the collected culture supernatant. All the collected supernatants were diluted 1:40 with 100 mM sodium acetate buffer (pH5.5) (*v/v*). Lane M: protein marker; lane 1: induction for 24 h; lane 2: induction for 48 h; lane 3: induction for 72 h; lane 4: induction for 96 h; lane 5: induction for 120 h; lane 6: induction for 144 h; lane 7: 0.05 mg/mL BSA; lane 8: 0.2 mg/mL BSA; lane 9: 0.3 mg/mL BSA

The Phy-producing capacity of strain 6c/HAC1 was evaluated further by high-density fermentation in a 10-L fermenter. Prepared liquid seeds were inoculated into the fermenter and the induction phase was started when the OD_600_ reached 230 (Fig. 4a). After induction for about 120 h, the highest observed recombinant phytase activity and protein content reached 35,032 U/mL and 9.58 g/L (Fig. 4a and b).

### Genetic stability

**Fig. 5:**
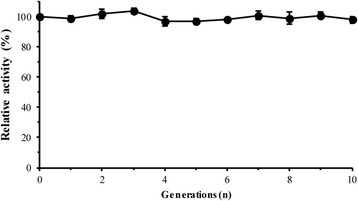
Changes in phytase activity of strain 6c/HAC1 after ten cultivations. The phytase activity of strain 6c/HAC1 after ten sub-cultivations with 96-h methanol induction. All activities were compared with the activity of the original clone, with GS115/HKA as the background sample

Figure 5 and Additional file 6 show that the phytase activity and *PHY* copy number of strain 6c/HAC1 was not significantly different after ten cultivations compared with the original colony. This indicated that the plasmids were highly stable during the sub-cultivations of the *P. pastoris* GS115 transformant at 30 °C in YPD medium. The genetic stability of the recombinant protein in this study was similar to that of the *A. niger* SK-27 *phyA* gene with the MF4I signal peptide expressed in *P. pastoris*, which retained 98 % of the phytase yield after ten cultivations [37].

## Discussion

All the strains of the signal peptide modifications have the same Phy gene copy number (Additional file 4), indicating that the insertion of a spacer peptide worked well with the *α*-factor and *PHY* and thus enhanced Phy secretion in *P. pastoris*. However, replacement of the reporter gene of *EGFP* by *PHY*, a deletion of the predicted third alpha helix of the *α*-factor, and replacement of the *α*-factor by the signal peptide of Dse4p did not work well, in contrast to the results of Lin-Cereghino et al. [12] and Liang et al. [14], which indicated that different signal peptides may have different secretion efficiencies with different proteins. The signal peptide determines whether co-translational translocation or post-translational translocation occurs for entrance to the ER and the secretion efficiency is related to the characteristics of the heterologous protein and the signal peptide [39].

Increasing gene copy numbers improved phytase expression obviously (Fig. 3a and Additional file 5). But when the copy number was increased further, there was a plateau effect (Fig. 3a and Additional file 5). It is plausible that direct correlation of expression level and gene copy number is not necessarily valid when the protein is directed to the secretory pathway. In that case, the production of a secreted protein will reach its limit at a specific gene copy number where further increases in transcription and translation due to the higher abundance of gene copies will not enhance the secretion process any further [40, 41].

Several studies have been conducted on high expression of phytase in *P. pastoris*. By codon usage optimization and α-factor modification, the phytase activity and protein content of *Peniophora lycii* phytase reached 10540 U/mL and 12.2 g/L in a 5-L fermenter [42]. *A. niger* SK-57 phytase was modified by using successive polymerase chain reactions and deleting intronic sequences, optimizing codon usage, and α-factor modification; phytase activity reached 865 U/mL and 6.1 g/L of protein content in a 2-L fermenter [37]. By changing transgene copy number, codon bias, sequence optimization, and temperature during expression, *Lillium longiflorum* phytase levels increased 1.2–20-fold [43]. However, most of these studies only solved a problem in one protein synthesis pathway, which will result in a bottleneck in another pathway. When larger amounts of polypeptides are processed in the ER, some are misfolded and degraded [21].

Here, overexpression of Hac1p, Pdi1p, and Ero1p enhanced the expression of Phy (Fig. 3a and c). Overexpression of Pdi1p and Ero1p increased disulfide bond formation activity [33]. Pdi1p behaves as a chaperone, inhibiting the aggregation of misfolded proteins [44]. Overexpression of Pdi1p enhanced expression of several heterologous proteins in *S. cerevisiae* [30, 45] and *P. pastoris* [46–48]. Overexpression of Ero1p enhanced the secretion of single-chain T-cell receptor (scTCR) in *S. cerevisiae* [49] and Fab fragment secretion in *P. pastoris* [48]. However, in our work, overexpression of Kar2p decreased the expression of Phy, similar to observations on *Schizosaccharomyces pombe* acid phosphatase expression in *S. cerevisiae* [50], but different from antithrombotic hirudin expression in *S. cerevisiae* [29] and Fab fragment secretion in *P. pastoris* [48]. The effects of overexpression of Kar2p seem to depend on the target recombinant protein. Overexpression of Hac1p enhances the expression of chaperones (e.g., Pdi1p) and affects many important cellular processes, including carbon metabolism, stress response and protein folding, enhancing protein secretion [51]. Thus Hac1p overexpression may solve the ER bottleneck in protein synthesis and increase the expression of heterologous proteins [27, 28]. Enhancement of protein folding in the ER can alleviate bottlenecks in the folding and secretion pathways during the overexpression of heterologous proteins in *P. pastoris* [21, 52].

## Conclusions

Using modifications of P_*AOX1*_ and the *α*-factor signal peptide, increased gene copy number, and overexpression of Hac1p to enhance folding and secretion of the protein in the endoplasmic reticulum, we have successfully increased the yield of *C. amalonaticus* CGMCC 1696 phytase in *P. pastoris* to 412 % of that for the original strain, Gs115/Phy. In a 10-L fed-batch fermenter, the phytase yield achieved was 9.58 g/L, with an activity of 35,032 U/mL; production of phytase can be directed towards different biocatalytic and feed additive applications.

## Additional files

Additional file 1: Figure S1. The construction of plasmids used in this study. A: Construction of the plasmids containing modified promoters and different signal peptides; B: Construction of the plasmids containing different copy numbers of *PHY*; C: Construction of the plasmids containing a *GAPDH* fragment; D: Construction of the plasmids containing different chaperone proteins and *HAC1*. (PDF 914 kb) Additional file 2: Table S1. Primers, vectors and strains used in this study. (PDF 254 kb) Additional file 3: Figure S2. Restriction enzyme digestion of plasmids containing two, four, and six expression cassettes. The plasmid 2c was digested using *Bgl*II, *Bam*HI, and *Kpn*2I, and the plasmids αE10, 4c, and 6c using *Bgl*II and *Bam*HI. The results of the restriction enzyme digestion were visualized using a 1 % (wet *w/v*) agarose gel. (PDF 111 kb) Additional file 4: Figure S3. Quantitative PCR assay of the Phy copy number in genomic DNA of recombinant yeast strains GS115/Phy, GS115/AOXm, GS115/SP-D, GS115/αE10 and GS115/α∆57-70. The threshold value (horizontal dashed line) was set at 0.2. The values indicate the average ± standard deviations from three independent qPCR experiments. (PDF 273 kb) Additional file 5: Figure S4. The phytase activity of strains containing different *PHY* copy numbers after induction for 96 h, using GS115/HKA as the background sample. (PDF 82 kb) Additional file 6: Figure S5. Quantitative PCR assay of the Phy copy number in genomic DNA of recombinant yeast strain 6c/HAC1 after ten sub-cultivations. The threshold value (horizontal dashed line) was set at 0.2. The values indicate the average ± standard deviations from three independent qPCR experiments. (PDF 291 kb)
