# Supplementary figures and images for: Combined strategies for improving expression of Citrobacter amalonaticus phytase in Pichia pastoris
Source: BMC Biotechnol. 2015 Sep 26;15:88. doi: 10.1186/s12896-015-0204-2 (PMC4584009; doi:10.1186/s12896-015-0204-2)

# Additional file 1. Supplemental figure 1.

A

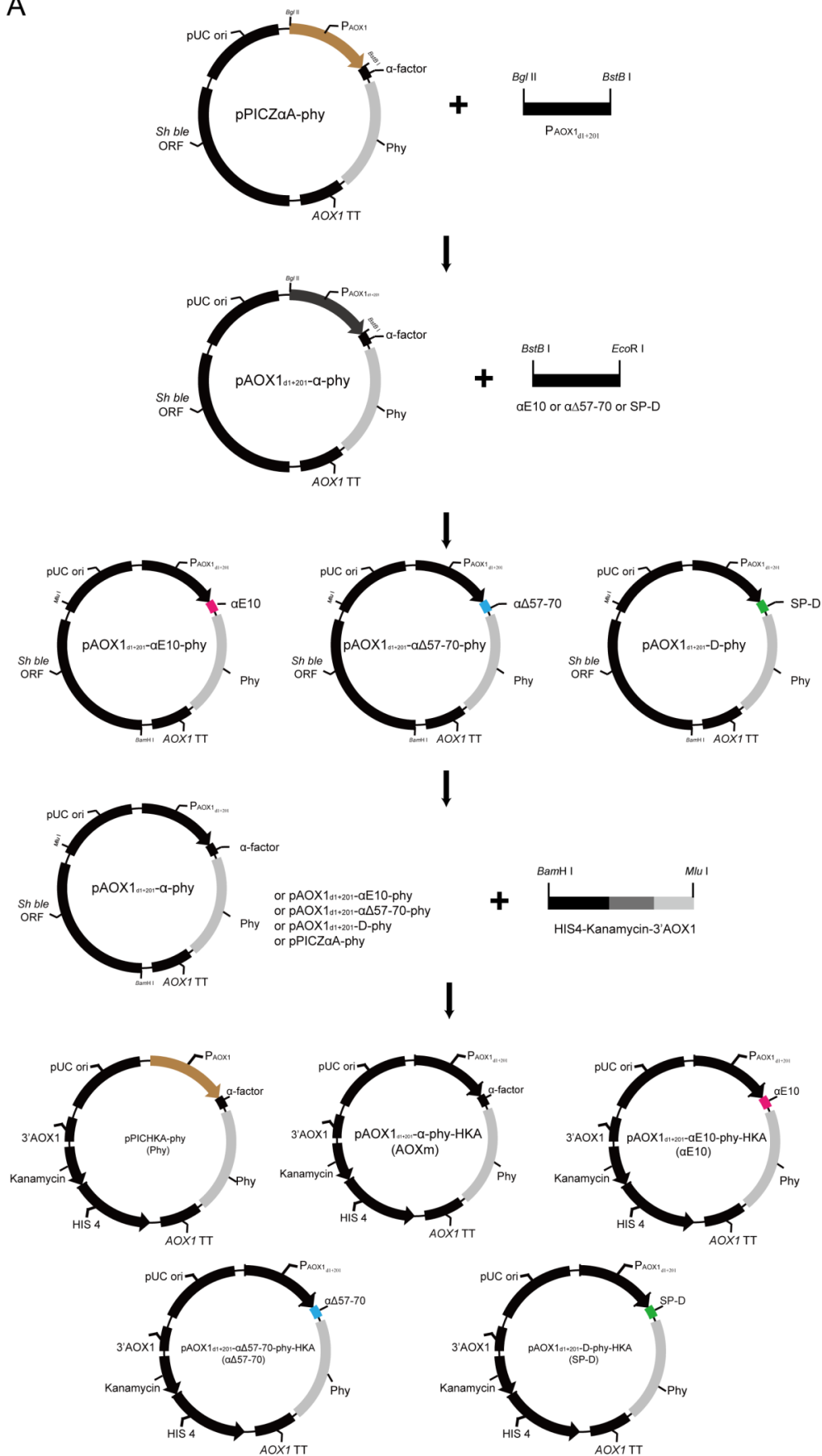

B

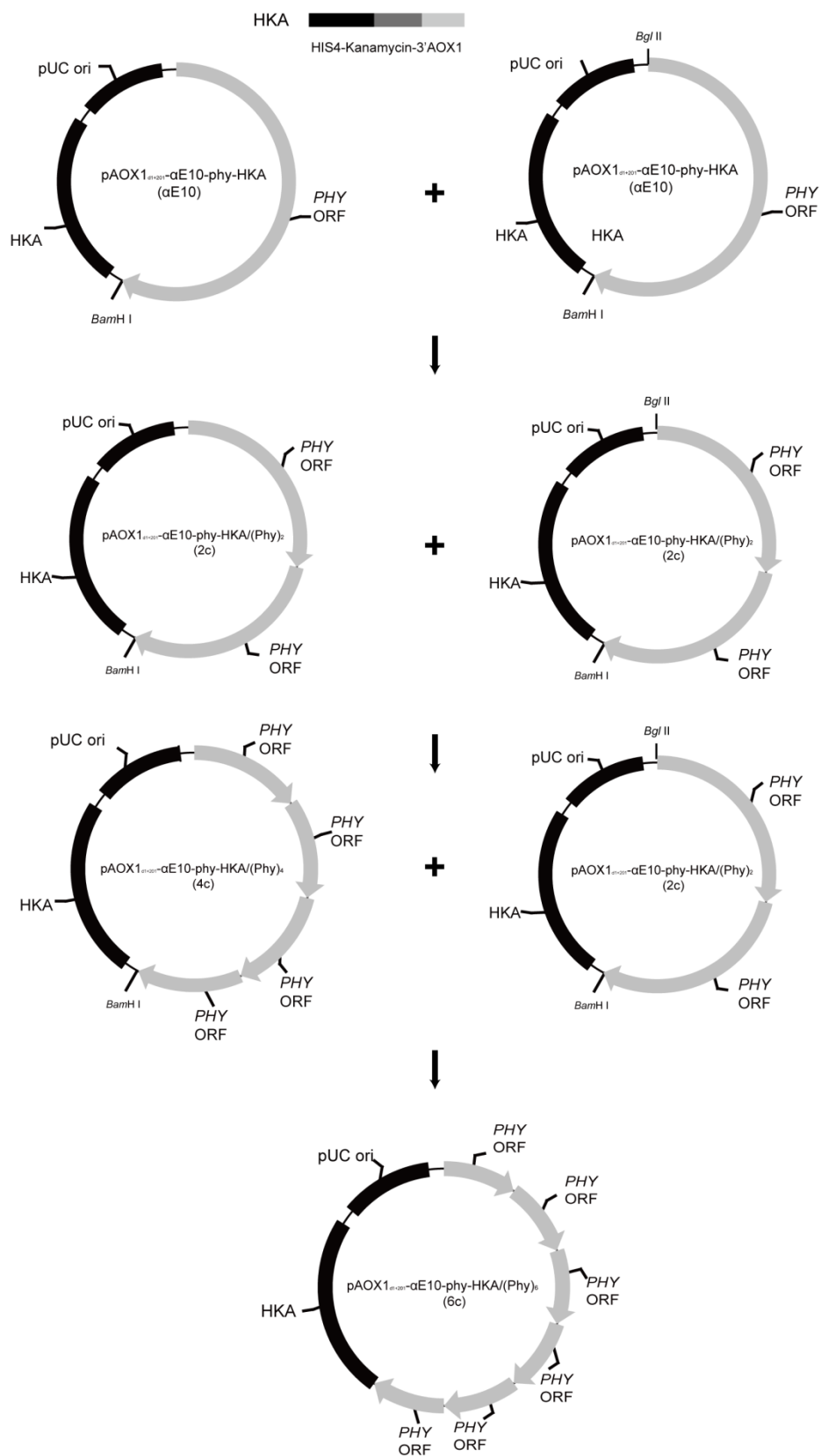

C

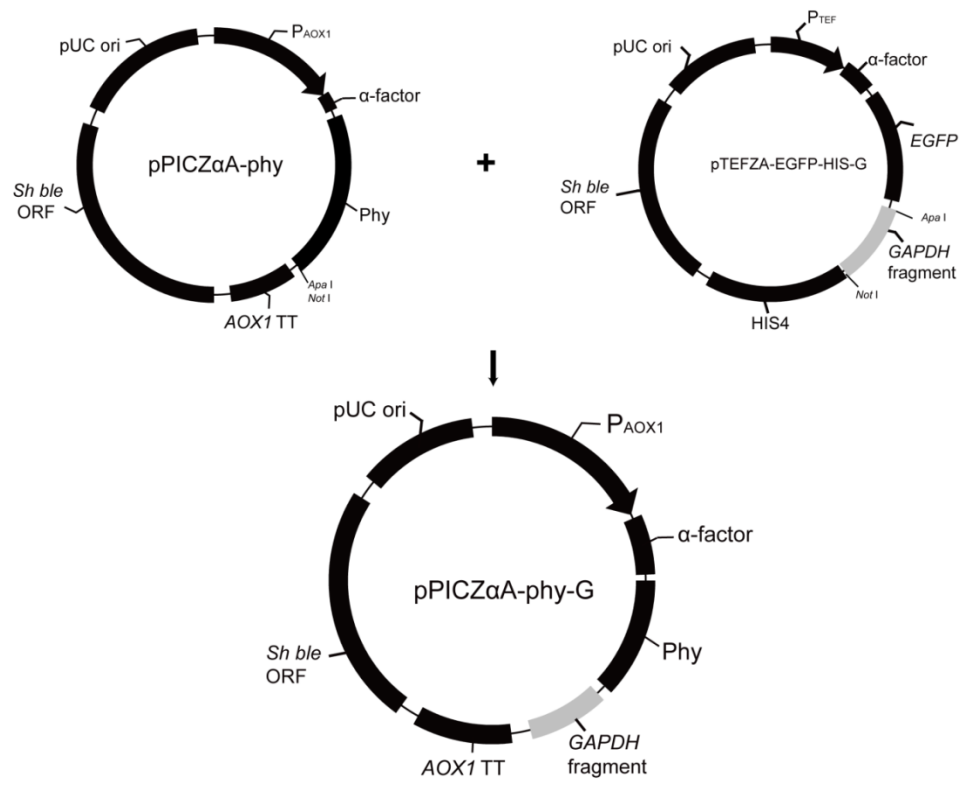

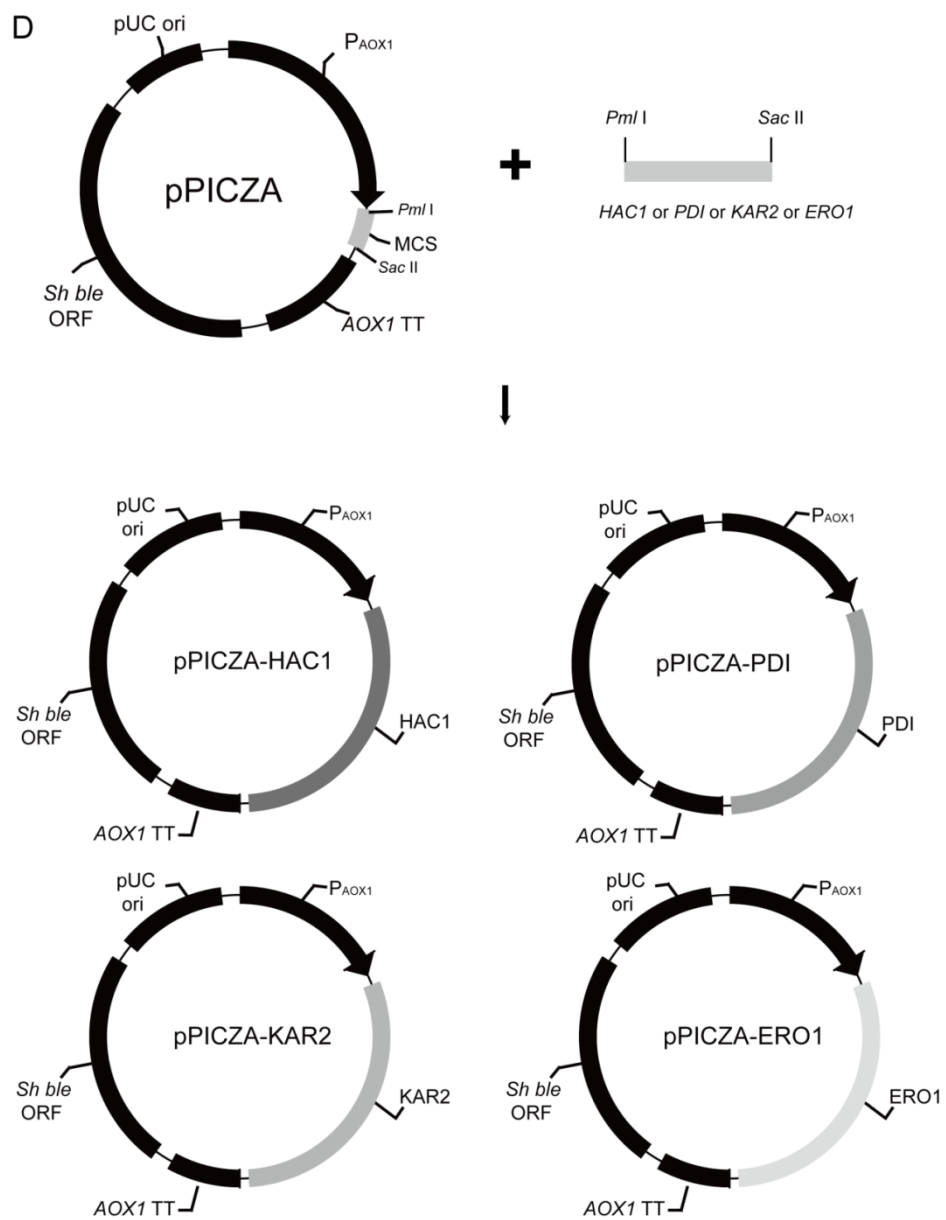

Supplement: Additional file 1: Figure S1. — The construction of plasmids used in this study. A: Construction of the plasmids containing modified promoters and different signal peptides; B: Construction of the plasmids containing different copy numbers of PHY; C: Construction of the plasmids containing a GAPDH fragment; D: Construction of the plasmids containing different chaperone proteins and HAC1. (PDF 914 kb) [file 12896_2015_204_MOESM1_ESM.pdf]

Additional file 3. Supplemental figure 2.

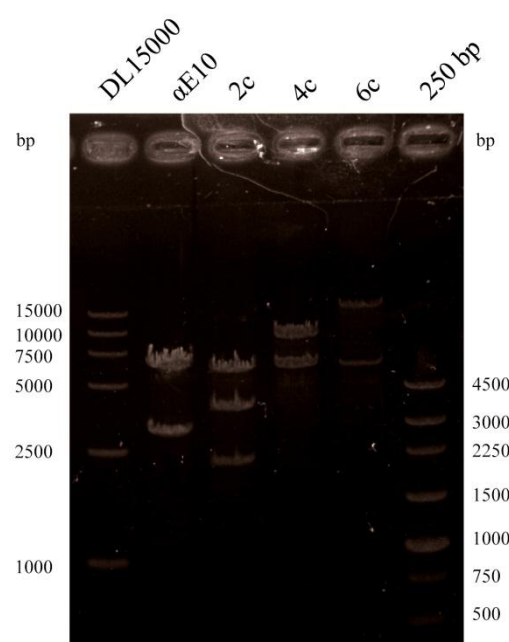

Supplement: Additional file 3: Figure S2. — Restriction enzyme digestion of plasmids containing two, four, and six expression cassettes. The plasmid 2c was digested using BglII, BamHI, and Kpn2I, and the plasmids αE10, 4c, and 6c using BglII and BamHI. The results of the restriction enzyme digestion were visualized using a 1 % (wet w/v) agarose gel. (PDF 111 kb) [file 12896_2015_204_MOESM3_ESM.pdf]

Additional file 4. Supplemental figure 3.

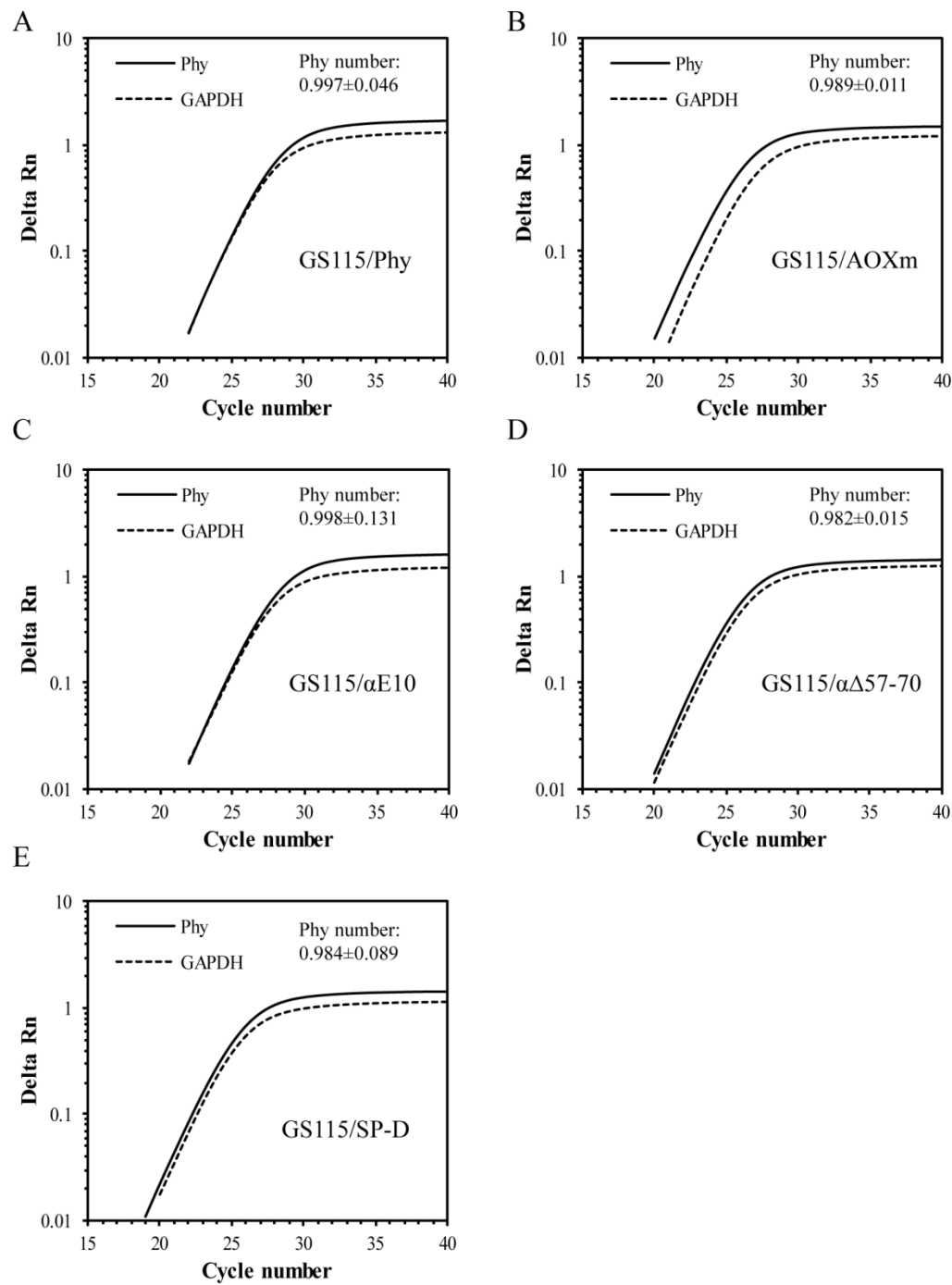

Supplement: Additional file 4: Figure S3. — Quantitative PCR assay of the Phy copy number in genomic DNA of recombinant yeast strains GS115/Phy, GS115/AOXm, GS115/SP-D, GS115/αE10 and GS115/α∆57-70. The threshold value (horizontal dashed line) was set at 0.2. The values indicate the average ± standard deviations from three independent qPCR experiments. (PDF 273 kb) [file 12896_2015_204_MOESM4_ESM.pdf]

**Additional file 5.** Supplemental figure 4.

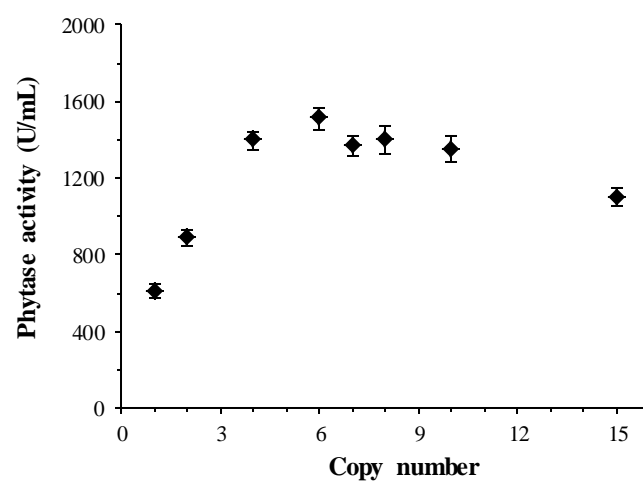

Supplement: Additional file 5: Figure S4. — The phytase activity of strains containing different PHY copy numbers after induction for 96 h, using GS115/HKA as the background sample. (PDF 82 kb) [file 12896_2015_204_MOESM5_ESM.pdf]

Additional file 6. Supplemental figure 5.

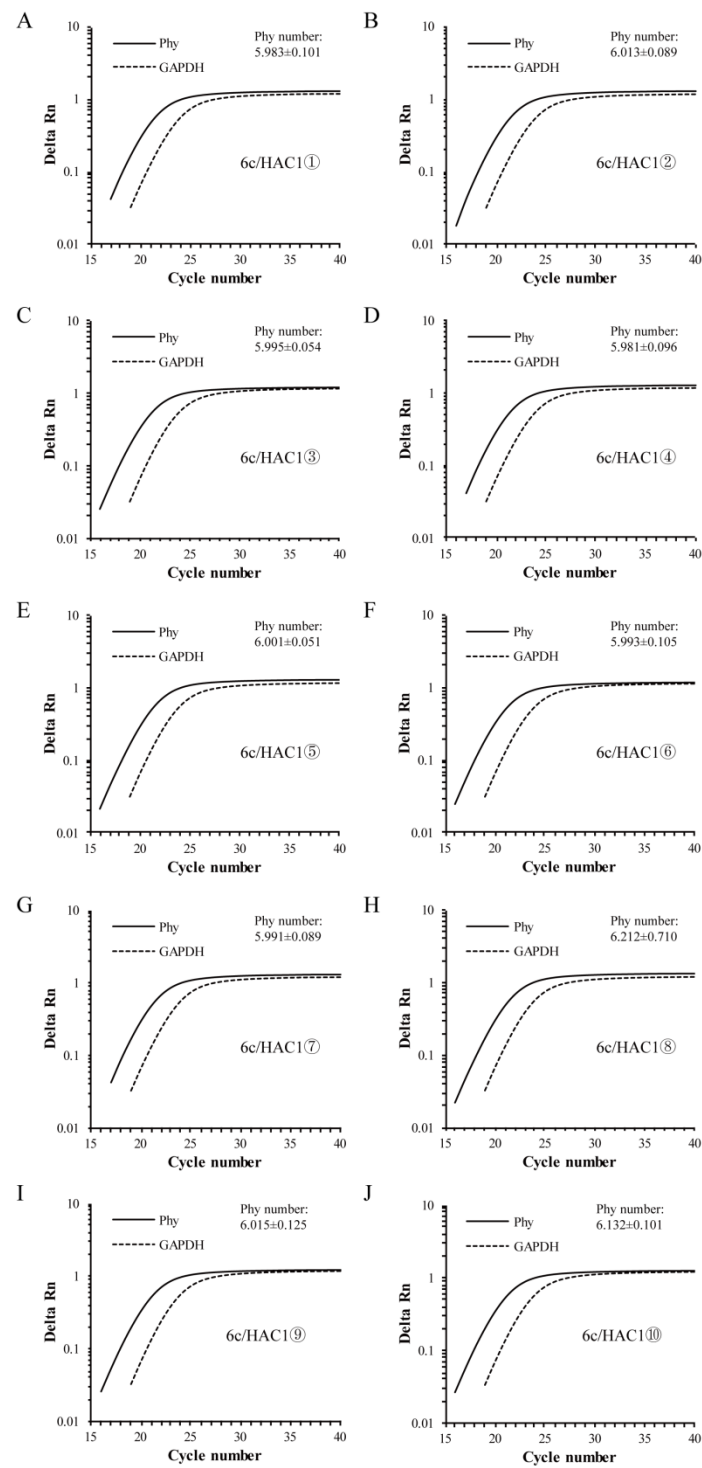

Supplement: Additional file 6: Figure S5. — Quantitative PCR assay of the Phy copy number in genomic DNA of recombinant yeast strain 6c/HAC1 after ten sub-cultivations. The threshold value (horizontal dashed line) was set at 0.2. The values indicate the average ± standard deviations from three independent qPCR experiments. (PDF 291 kb) [file 12896_2015_204_MOESM6_ESM.pdf]
