# Supplementary material for: Combined strategies for improving expression of Citrobacter amalonaticus phytase in Pichia pastoris
Source: BMC Biotechnol. 2015 Sep 26;15:88. doi: 10.1186/s12896-015-0204-2 (PMC4584009; doi:10.1186/s12896-015-0204-2)
Supplement: Additional file 2: Table S1. — Primers, vectors and strains used in this study. (PDF 254 kb) [file 12896_2015_204_MOESM2_ESM.pdf]

Additional file 2. Supplemental table 1

The restriction sites in the primer sequences are underlined.

	Primer sequences (5'-3') or a short description of the plasmids	Endonucleases
AOX F	AGC<u>AGATC</u>TAACATCCAAAGACGAAAGGTTGA	*Bgl*II
AOX R	ACA<u>AAGCTT</u>GTCAATTGGAACCAGTCGCAATT	*Bst*BI
D1 F	TCCTCAACACCCACTTTAGGCTACTAACACCATGACTTTATTAGCCTGTC	
D1 R	GTGTTAGTAGCCTAAAGTGGGTGTTGAGGAGAAGAGGAGT	
201-2xfw	GCTGATAGCCTAACGTTCATGATCAAAATTTCATGATCAAAATTTAACTGTTC	
201-2xrv	GTTAAATTTTGATCATGAAATTTTGATCATGAACGTTAGGCTATCAGCAGTATTC	
SP-D1	CGTG<u>TTCGAA</u>ATGTCATTCTCTTCCAAC	*Bst*BI
SP-D2	CAGC<u>GAATTC</u>TCCACTGACTATATTGGTC	*Eco*RI
SP-M1	CCG<u>TTCGAA</u>ACGATGAGATTTCCTTCAA	*Bst*BI
SP-M2	CTA<u>GAATTC</u>CTTGGGCTCAGCCTCAGCTTCAGCCTCCTCAGCTTCAGCCTCTCT	*Eco*RI
SP-Δ57-70-1	TTTGCCATTTTCCGCCAGCATTGCTGCTAAAGAAGAAGGGGTA	
SP-Δ57-70-2	CAGCAATGCTGGCGGAAAATGGCAAAACAGCAACATCGA	
SP-Δ2	CACGT<u>GAATTC</u>AGCTTCAGCCTCTC	*Eco*RI
PDI-1	GGCG<u>CACGTG</u>ATGAAAATATTAAGTGCATTGCTTC	*Pml*I
PDI-2	TAG<u>CCGCGG</u>AGTAGCTCTTGGTGTAATAACTGGG	*Sac*II
KAR2-1	TGCA<u>CACGTG</u>ATGCTGTCGTTAAAACCATCTTG	*Pml*I
KAR2-2	ATCG<u>CCGCGG</u>TACAACTCATCATGATCATAGTCATA	*Sac*II
ERO1-1	GCCGA<u>CACGTG</u>ATGAGGATAGTAAGGAGCGTAGCTA	*Pml*I
ERO1-2	CGTCG<u>CCGCGG</u>TACAAGTCTACTCTATATGTGGTATCT	*Sac*II
RT-G1	GTCGGGACACGCCTGAAACT	

RT-G2		CCACCTTTTGGACCCTATTGAC
RT-Phy1		TGGTTGGGGTAGAATCAC
RT-Phy2		TGCTTCTGAGGAGGATGA
**Plasmids**	**Plasmids (abbreviation)**	
pPICZA		Invitrogen
pPICHKA	HKA	To offer the *his4* and kanamycin resistance ORF
pPICZαA-phy		To offer the *phy*
pPICHKA-phy	Phy	To replace the *Sh ble* ORF with *his4* and kanamycin resistance ORF
pAOX1$_{d1+201}$-α-phy		To combine cis-acting elements with the promoter *AOX1*
pAOX1$_{d1+201}$-α-phy-HKA	AOXm	To replace the *Sh ble* ORF with *his4* and kanamycin resistance ORF
pAOX1$_{d1+201}$-αE10-phy		To add 10-residues spacer peptide (EEAEAEAEPK) after α-factor prepro-signal
pAOX1$_{d1+201}$-αE10-phy-HKA	αE10	To replace the *Sh ble* ORF with *his4* and kanamycin resistance ORF
pAOX1$_{d1+201}$-αΔ57-70-phy		To delete predicted 3rd alpha helix of the α-factor
pAOX1$_{d1+201}$-αΔ57-70-phy-HKA	αΔ57-70	To replace the *Sh ble* ORF with *his4* and kanamycin resistance ORF
pAOX1$_{d1+201}$-D-phy		To replace the α-factor with signal peptide of Dse4p
pAOX1$_{d1+201}$-D-phy-HKA	SP-D	To replace the *Sh ble* ORF with *his4* and kanamycin resistance ORF
pPICZA-αE10-HKA/(Phy)$_2$	2c	To increase *phy* expression cassettes
pPICZA-αE10-HKA/(Phy)$_4$	4c	To increase *phy* expression cassettes
pPICZA-αE10-HKA/(Phy)$_6$	6c	To increase *phy* expression cassettes
pTEFZA-EGFP-HIS-G		To offer the *gapdh* gene fragment
pPICZαA-phy-G		To detect *phy* gene copy number
pPICZA-HAC1		To overexpress Hac1p
pPICZA-PDI		To overexpress Pdi1p
pPICZA-KAR2		To overexpress Kar2p
pPICZA-ERO1		To overexpress Ero1p
**Strains**	**Strains (abbreviation)**	

Escherichia coli TOP10F′		Invitrogen
Pichia pastoris GS115		Invitrogen
GS115/ pPICHKA	GS115/ HKA	For background control
GS115/ pPICHKA-phy	GS115/Phy	For secreted expression of phytase using *AOX1* promoter and α-factor
GS115/ pAOX1$_{d1+201}$-α-phy-HKA	GS115/AOXm	For secreted expression of phytase using *AOX1*$_{d1+201}$ promoter and α-factor
GS115/ pAOX1$_{d1+201}$-αE10-phy-HKA	GS115/αE10	For secreted expression of phytase using *AOX1*$_{d1+201}$ promoter and α-factor with 10-residues spacer peptide (EEAEAEAEPK)
GS115/ pAOX1$_{d1+201}$-αΔ57-70-phy-HKA	GS115/αΔ57-70	For secreted expression of phytase using *AOX1*$_{d1+201}$ promoter and α-factor with deletion predicted 3rd alpha helix of the α-factor
GS115/ pAOX1$_{d1+201}$-D-phy-HKA	GS115/SP-D	For secreted expression of phytase using *AOX1*$_{d1+201}$ promoter and signal peptide of Dse4p
GS115/ pPICZA-αE10-HKA/(Phy)$_2$	GS115/2c	For secreted expression of phytase using 2 *phy* expression cassettes
GS115/ pPICZA-αE10-HKA/(Phy)$_4$	GS115/4c	For secreted expression of phytase using 4 *phy* expression cassettes
GS115/ pPICZA-αE10-HKA/(Phy)$_6$	GS115/6c	For secreted expression of phytase using 6 *phy* expression cassettes
GS115/ pPICZA-αE10-HKA/(Phy)$_n$	GS115/nc	For secreted expression of phytase using n *phy* expression cassettes
pPICZA-αE10-HKA/(Phy)$_6$/ pPICZA-HAC1	6c/HAC1	For secreted expression of phytase using 6 *phy* expression cassettes and overexpression of Hac1p
pPICZA-αE10-HKA/(Phy)$_6$/ pPICZA-PDI	6C/PDI	For secreted expression of phytase using 6 *phy* expression cassettes and overexpression of Pdi1p
pPICZA-αE10-HKA/(Phy)$_6$/ pPICZA-KAR2	6c/KAR2	For secreted expression of phytase using 6 *phy* expression cassettes and overexpression of Kar2p
pPICZA-αE10-HKA/(Phy)$_6$/ pPICZA-ERO1	6C/ERO1	For secreted expression of phytase using 6 *phy* expression cassettes and overexpression of Ero1p
